# Experimental and Numerical Study on Uniaxial Compression Failure of Concrete Confined by Nylon Ties

**DOI:** 10.3390/ma15092975

**Published:** 2022-04-19

**Authors:** Hui Wang, Shichang Shang, Hang Zhou, Cheng Jiang, Hongyuan Huai, Zhichao Xu

**Affiliations:** 1 College of Resources, Shandong University of Science and Technology, Tai’an 271019, China; sschang12@163.com (S.S.); zhouhang0504@163.com (H.Z.); huaihongyuan@163.com (H.H.); xzchao5217@163.com (Z.X.); 2 College of Civil Engineering and Architecture, Shandong University of Science and Technology, Qingdao 266590, China; 3 Xuzhou Shouchuang Water Co., Ltd., Xuzhou 221000, China; 13814433385@163.com

**Keywords:** nylon ties, structural reinforcement engineering, active confinement, uniaxial compression, numerical simulation

## Abstract

The developments in the cisvil engineering fields have led to an increased demand for structural reinforcements. Therefore, designing an effective, green, convenient, and low-cost reinforcement method is considerably important. Nylon ties have high strength and good heat resistance, and they can retain good tensile properties after high-temperature cooling. Further, they are inexpensive and can be recycled. Thus, they are considered suitable for structural reinforcement engineering. In this study, the core concrete was reinforced by the active confinement of prestressed nylon ties. A uniaxial compression test was performed to evaluate the reinforcement effect of the preload generated by the high-temperature cooling of the tie rod on the core concrete. The results show that nylon ties can effectively improve the mechanical properties of the core concrete. Combined with numerical simulation technology, a damage model of a confined concrete column (CC) was established, and the damage evolution law of CC under uniaxial axial compression was analyzed. Combined with numerical simulation and experimental research, the effectiveness of nylon tie reinforced concrete and the reliability of the damage model were verified, providing a reference for research on engineering reinforcement.

## 1. Introduction

The reinforcement of engineering structures is a major challenge in civil engineering. The steel pasting method can effectively improve the structural stiffness. However, steel materials are heavy, easily corroded, and inconvenient to construct [1,2,3]. Moreover, steel with high hardness is unsuitable for concrete columns with irregular cross-sectional shapes [4]. Fiber-reinforced polymer (FRP) materials are light, have considerable mechanical strength, and have been used in the early stages of the aerospace engineering field. To date, many scholars have used FRP to study the restraint of concrete structures and have made many achievements [5,6,7,8]. However, for FRP materials, the cost of carbon fiber FRP (CFRP) is high, aramid FRP is expensive and has a high relaxation rate, and the tensile strength of glass FRP is low [3].

Polymer materials include thermoplastics and thermosetting materials [4]. Among the thermosetting materials, thermosetting phenolic resin, which is commonly used in electrical products, easily absorbs water and has a good insulation performance [9,10]. Phenolic epoxy resin has strong adhesion, is cured in low-temperature and low-pressure environments, and is commonly used as an adhesive [11,12]. Polyester fiber is an excellent textile material with low strength reduction at high temperatures, similar elasticity to wool, and good shape retention [13,14]. Compared to thermosetting, thermoplastic materials can be remolded after heating. Moreover, thermoplastic materials are easily deformed after heating and their strength increases after cooling [15]. Polyvinyl chloride is a thermoplastic material that is non-toxic and resistant to high temperatures. Hard materials can be used in downpipes and hard water pipes. Soft materials can be used in cables, and auto parts. Polyethylene (PE) has good processing performance, is easy to burn, and can be made into a film. Acrylonitrile butadiene styrene (ABS) resin is used to prepare various parts of machinery with high strength and toughness. Polyamide (PA), also known as nylon, is a semicrystalline crystal material that can maintain good stiffness and strength even in a high-temperature environment. The main chain of PA contains many repeated PA groups—[NHCO] [16]. PA has good impact properties and tensile strength, and is widely used in various mechanical and electrical parts. Compared to other polymer materials, PA materials are widely used in engineering because of their good mechanical properties [17,18,19,20].

When a structure is in a state of multidirectional compression, the lateral pressure on the confined concrete can effectively limit the expansion of the microcracks in the structure. This can effectively restrain the transverse and longitudinal deformations, as well as effectively improve the compressive strength and ductility of concrete structures. There are two types of concrete confinement methods: active and passive [21,22,23,24]. In contrast to the passive confined form, the active confined pattern can effectively avoid the phenomenon of stress hysteresis and take full advantage of the confinement effect of the materials. There are several methods of applying an active confinement force. Moghaddam et al. adopted the method of post-tensioned metal strips to enable the concrete column to bear the initial lateral pressure before the axial load [25]. Liu et al. applied a prestress to FRP laminates by using a manual hydraulic jack [3]. The temperature method is suitable for materials with a low melting point that are easy to soften. After heating, the material shrinks and deforms to provide an initial lateral pressure to the component [4,26]. This is a simple and inexpensive method.

In summary, each constraint case has its own characteristics, but effective, environmentally friendly, cheap, and convenient constraint methods have been rarely reported. Moreover, most cases are limited to the study of the constitutive relationship of confined concrete, and there the research on the damage evolution law of confined concrete is scarce. Therefore, a novel confined concrete with heated nylon ties is proposed. In this study, the active confined stress due to heated nylon ties was investigated to determine whether the nylon ties were effective in enhancing the mechanical properties of the core concrete, to provide an effective, green, and low-cost reinforcement method for civil engineering projects. The damage model of confined concrete columns (CCs) was established, and the damage law of uniaxial-compression CCs and the breakage of nylon ties were analyzed. The results provide a reference for engineering structural reinforcements.

## 2. Laboratory Tests

### 2.1. Experimental Study on Mechanical Properties of Nylon Ties

**Figure 1:**
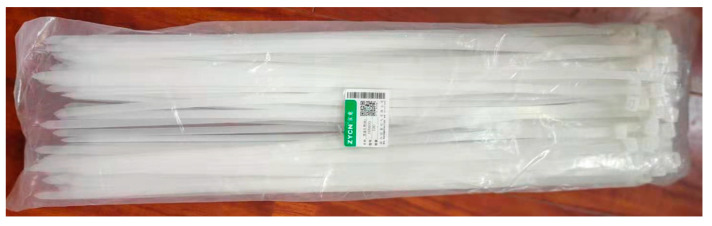
Nylon ties.

Nylon ties (Figure 1) have the following advantages: excellent mechanical properties, high mechanical strength, good toughness, high melting point, maintenance of good stiffness and strength in a high-temperature environment, good elasticity, and fatigue resistance. They also have a low cost and can be recycled. Therefore, they are suitable for use in structural reinforcement engineering.

The tie-wraps were heated in an electric blast drying oven at 80 °C, 100 °C, and 120 °C. After the heated nylon ties were cooled, a pre-tightening test with a load ring and tensile test were conducted.

**Figure 2:**
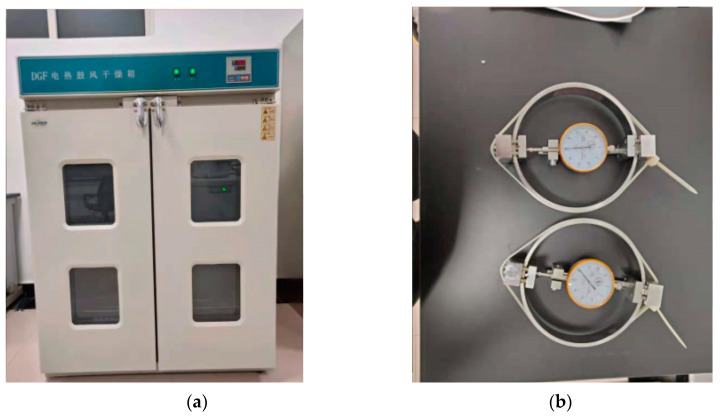

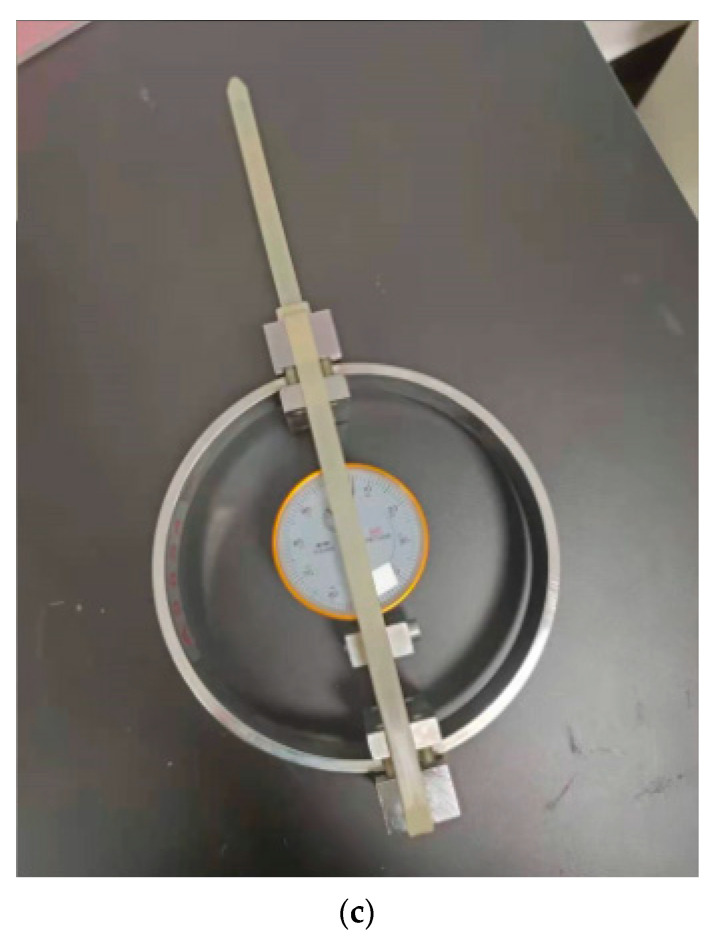
Prestressing test: (**a**) electric blast drying oven (Shanghai Yuejin Experimental Instrument Factory, Shanghai, China), (**b**) circumferential winding, (**c**) radial winding.

The heating time was 3 h (Figure 2a). The lateral stress of the heated nylon ties after cooling was measured using a load ring with a diameter of 150 mm. During the tests, the ties were wound along the circumferential and radial directions of the load ring (Figure 2b,c). After the ties were removed from the oven, they cooled and deformed rapidly. Thus, they were wound on the load ring within 10 s.

**Figure 3:**
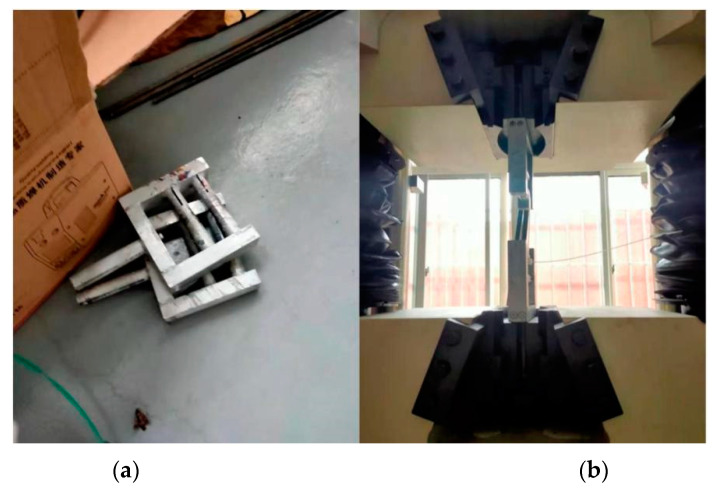
Tensile test: (**a**) fixture, (**b**) testing machine (Xinte Testing Machine Co., Changchun, China).

The mechanical properties of the nylon ties were determined via tensile testing. The screw clamp of the tensile testing machine in the laboratory was difficult to use for winding the ties. Binding was wrapped using a customized winding fixture (Figure 3).

### 2.2. Uniaxial Compression Test

**Figure 4:**
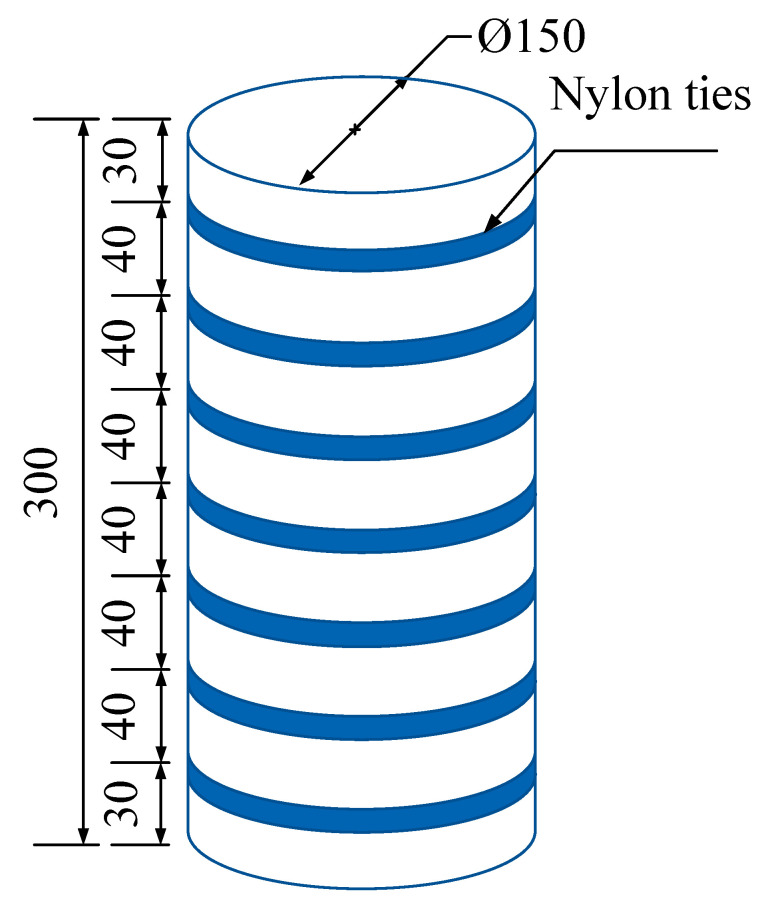
Confinement scheme (mm).

**Figure 5:**
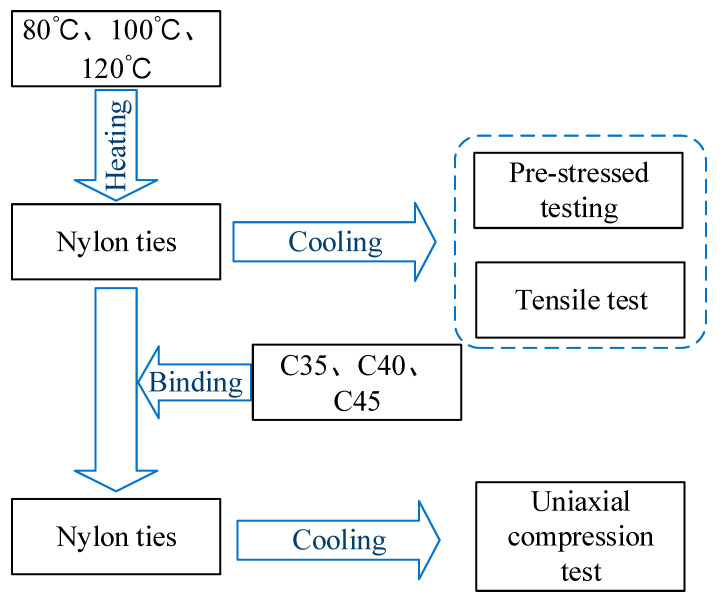
Test process.

A total of 12 specimens were used in this test (Table 1). The effects of the concrete strength grade and heating temperature on the reinforcement effect were considered. The confinement scheme of the specimens is shown in Figure 4. The test process is shown in Figure 5.

## 3. Establishment of Finite Element Model

Taking Y35-100 and Y35 as examples, to use the numerical method combined with the test results to explore the damage evolution law of concrete, a uniaxial compression test was conducted. Therefore, the uniaxial compression model was selected for the simulation, which did not consider other types of deformation. The Y35-100 model size was as follows: the tie thickness was 1 mm and the model element type was C3D8R, which was divided into 68,508 elements. The Y35 concrete model size was ϕ150 mm × 300 mm. Hexahedral elements and sweep methods were used for mesh generation. The element type was C3D8R, and 52,800 elements were used.

**Figure 6:**
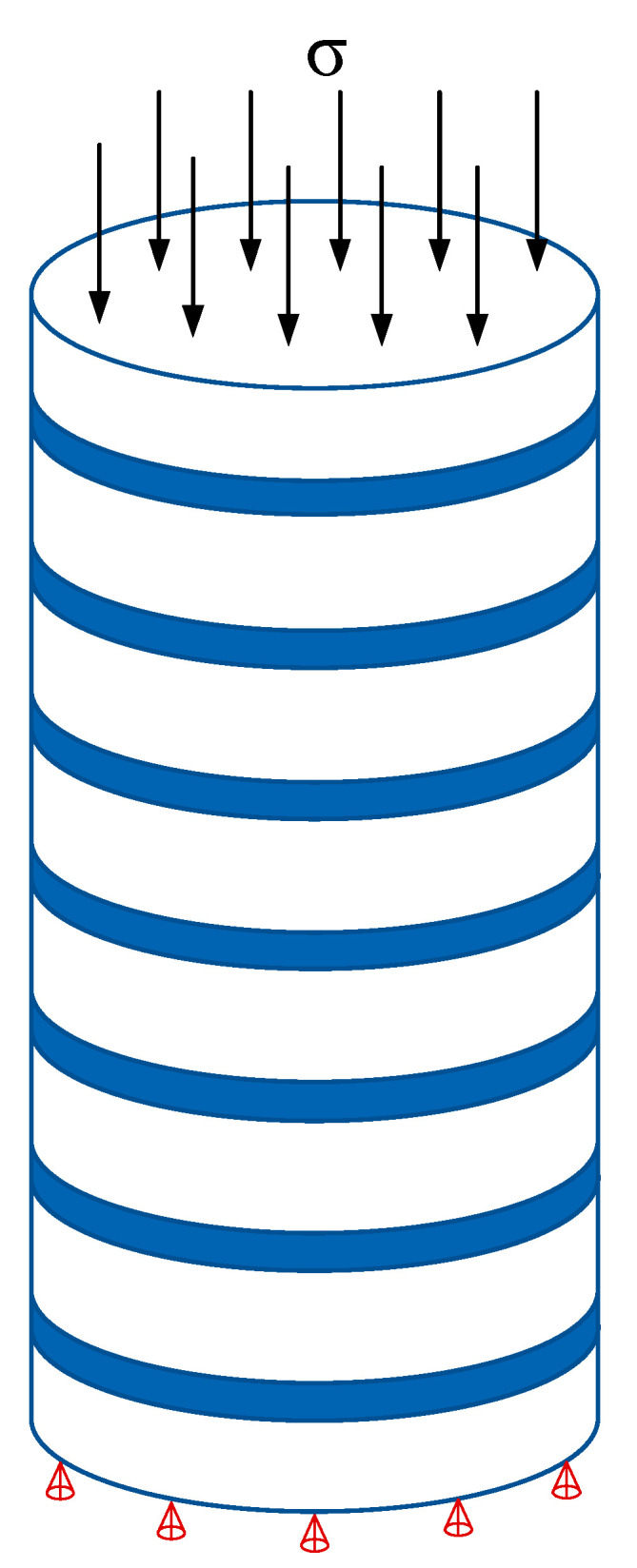
Force model.

In the uniaxial compression simulation, the bottom of the model was constrained vertically, and the upper surface was compressed by displacement control. The maximum displacement of the loading was 30 mm and the loading rate was controlled at 0.083 mm/s (Figure 6).

### 3.1. Damage Plastic Model of Concrete

**Figure 7:**
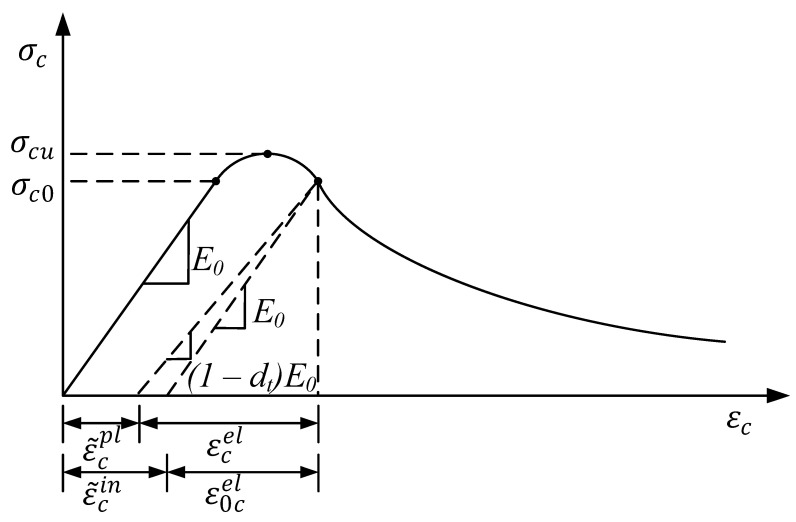
Compressive constitutive model of concrete [27].

The hardening process was divided into strengthening and softening stages. When the concrete stress reached the peak stress $\sigma_{cu}$, the material entered the softening stage. In the softening stage, the concrete material was damaged, and the most intuitive conclusion that can be drawn from the figure is that the unloading stiffness of the material decreased to $\left(1-d_{t}\right)E_{0}$ (Figure 7).

Nylon ties have a certain strengthening effect on concrete. In addition to the strength of the concrete core, this is mainly determined by the size of the confined force; that is, the stronger the confined force, the greater the compressive strength of the concrete.

The strength formula of CC is as follows [28]:

(1) $$\sigma_{cc}^{\prime }=\left(\sigma_{co}^{\prime },\sigma_{l}\right),$$

where $\sigma_{cc}^{\prime }$ and $\sigma_{co}^{\prime }$ are the compressive strengths of CC and unconfined concrete (UC), respectively, and $\sigma_{l}$ is the confinement stress (obtained from Section 2.1).

**Figure 8:**
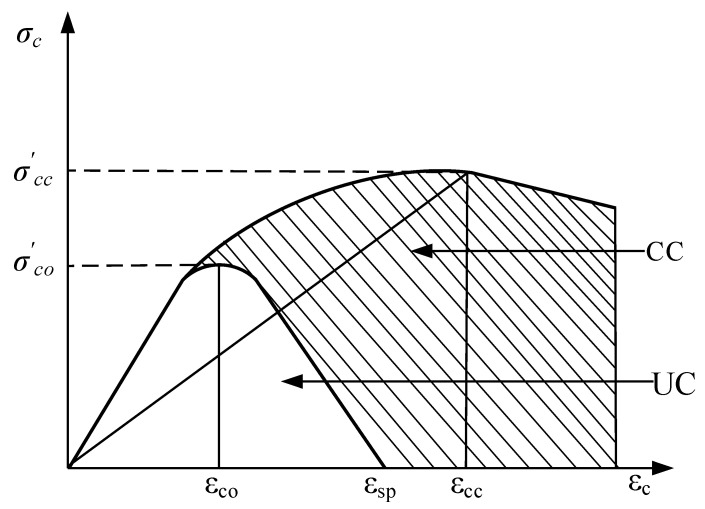
Stress-strain curves of UC and CC [28].

The stress-strain curve of confined concrete can be expressed by the following formulae (Figure 8):

(2) $$\sigma_{cc}^{\prime }=\left(\sigma_{co}^{\prime },\sigma_{l}\right),$$

(3) $$x=\frac{\varepsilon_{c}}{\varepsilon_{cc}},$$

(4) $$\varepsilon_{cc}=\varepsilon_{co}\left[1+5\left(\frac{\sigma_{cc}^{\prime }}{\sigma_{co}^{\prime }}-1\right)\right],$$

where $\varepsilon_{co}$ is the peak compressive strain of UC.

(5) $$r=\frac{E_{c}}{E_{c}-E_{sec}},$$

(6) $$E_{c}=5000\sqrt{\sigma_{co}^{\prime }},$$

(7) $$E_{sec}=\frac{\sigma_{cc}^{\prime }}{\varepsilon_{cc}^{\prime }},$$

(8) $$\sigma_{cc}^{\prime }=\sigma_{co}^{\prime }\left(-1.254+2.254\sqrt{1+\frac{7.94fl^{\prime }}{\sigma_{co}^{\prime }}}-2\frac{fl^{\prime }}{\sigma_{co}^{\prime }}\right),$$

(9) $$fl^{\prime }=\sigma_{l}S,$$

where S is the cross-sectional area of nylon ties.

To simulate the damage process of concrete under uniaxial compression, a damage model of concrete was established using the element damage function provided by ABAQUS. The damage criterion of concrete is:

(10) $${\bar{\varepsilon }}_{c}^{pl}\geq 0.05.$$

### 3.2. Failure Model of Nylon Ties

After the concrete bears the axial load, the tie bears the tensile force exerted by the concrete core. When the tensile stress reaches a maximum value, the tie is destroyed. The maximum tensile stress failure criterion is embedded in the VUMAT subroutine. The failure element deletion algorithm was introduced to simulate the failure process of ties.

The maximum tensile stress criterion of the element is as follows:

(11) $$f=\left|\sigma_{1}\right|-\sigma_{t},$$

where $\sigma_{1}$ and $\sigma_{t}$ are the maximum principal stress and critical tensile stress of the element, respectively.

**Figure 9:**
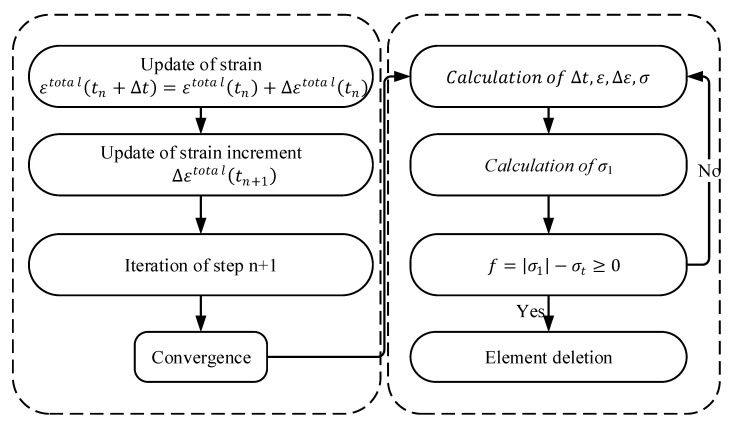
Flowchart of fracture damage to nylon ties.

Under the action of an external force, when the stress state of an element of the tie model satisfies the criterion condition of Equation (11), the element is identified as a failure and removed from the iterative calculation of the next load step. The iterative calculation is repeated until the residual error of the internal force of all elements meet the convergence criterion. When the calculation is terminated, the stress and deformation results of the element are output [29]. The value of $\sigma_{t}\;$ is taken from the tensile test parameters in Section 2.1. A flowchart of fracture damage to nylon ties is shown in Figure 9.

## 4. Experimental Results

### 4.1. Analysis of Mechanical Properties of Nylon Ties

**Figure 10:**
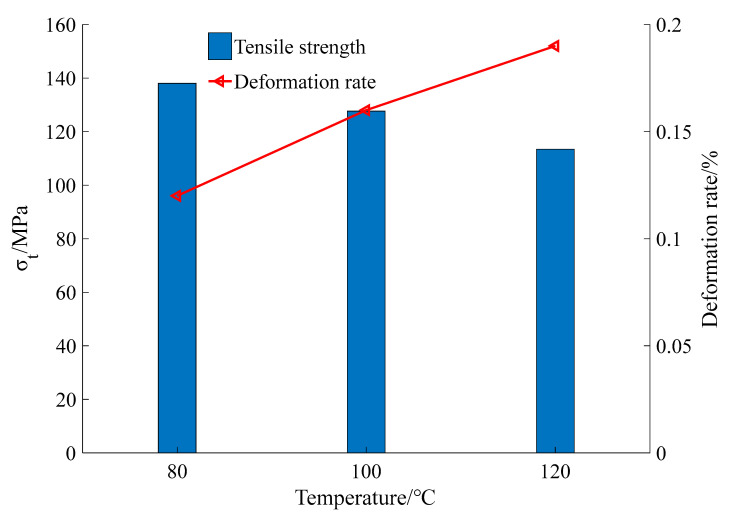
Properties of nylon ties.

The glass transition temperature of nylon is 55–58 °C, and the heating temperature is 80–120 °C. With an increase in temperature, the shrinkage deformation increased; however, the toughness decreased, leading to a decrease in the tensile strength. The cooling deformation increased with increasing temperature (Figure 10, Table 2).

**Figure 11:**
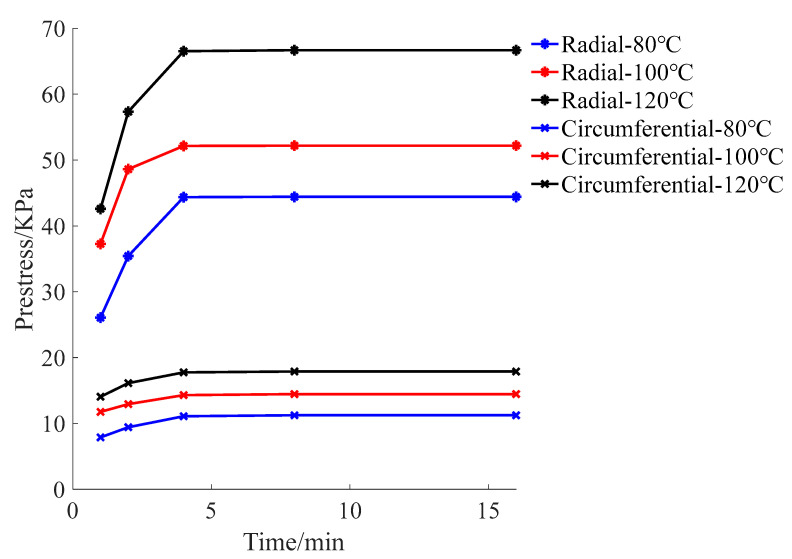
Prestress curve.

After taking the ties out of the oven, the ties quickly cooled. The cooling rate was the fastest at 0–1 min. After 4 min, no cooling deformation was observed. The higher the temperature, the greater the prestress produced by the cooling shrinkage of the ties. When the stress area of radial winding was smaller than that of circumferential winding, the prestress produced by radial winding was larger than that produced by circumferential winding at the same temperature (Figure 11, Table 3 and Table 4).

### 4.2. Analysis of Confined Concrete under Uniaxial Compression

**Figure 12:**
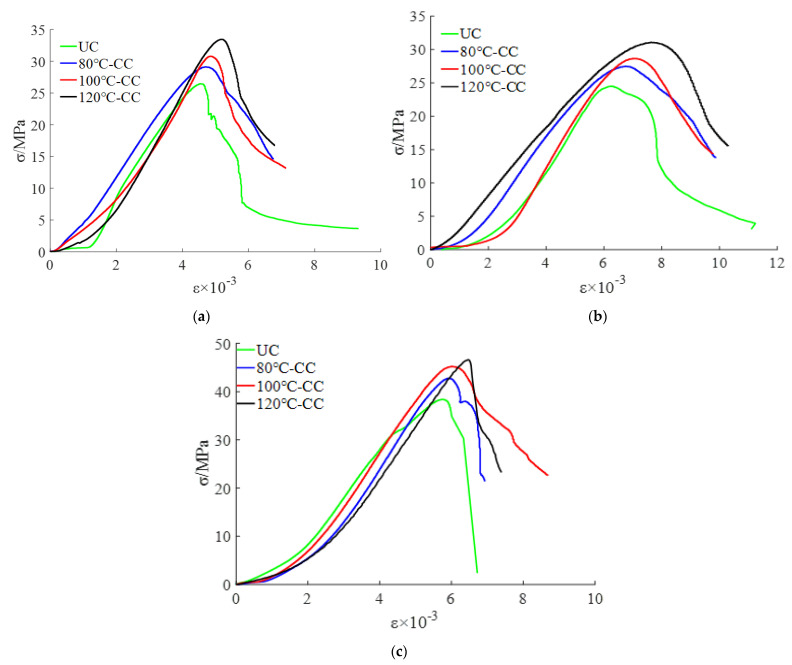
Stress-strain curves of concrete columns: (**a**) C35 concrete, (**b**) C40 concrete, (**c**) C45 concrete.

The stress-strain curve of concrete under uniaxial compression reflects the process of material failure and can reflect the important performance parameters of the materials. In this test, 12 groups of specimens were used to measure the uniaxial compression stress-strain curves of CC with different concrete strength grades and heating grades of the nylon ties, as shown in Figure 12.

The compressive strength of the CC was significantly higher than that of the UC. The effect of the 120 °C confinement scheme (120 °C-CC) was the best. The average peak strain of UC was 4.72 × 10^−3^, and the average peak stress was 28.64 MPa. The average peak stresses of 80 °C-CC, 100 °C-CC, and 120 °C-CC were 31.64 MPa, 33.99 MPa, and 35.83 MPa, which were 9.46%, 15.73%, and 20.06% higher than that of UC, respectively. The reason for this is that confined concrete can increase the energy damage release rate required for concrete microcrack propagation. When the confined concrete bears an axial load, the volume of the core concrete expands and the cross-sectional area increases. The nylon ties bear the tensile force and the core concrete bears the lateral binding force imposed by the ties to restrain the cracking degree of the core concrete. According to the force balance relationship, the crack suppression force provided by the tie, σ, can offset part of the fracture stress.

The initial elastic modulus of the confined concrete was positively correlated with the heating grade of the tie. The higher the prestress grade, the greater the initial elastic modulus of the concrete, the greater the structural stiffness, and the stronger the ability of the structure to resist deformation.

In the early stage of loading, owing to the existence of prestress, when the concrete block was under pressure, the prestress restrained the growth of the internal force of the block. The cracks appeared late, and volume expansion slowly developed.

When the applied stress reached the peak stress, the surface cracks of the concrete columns rapidly expanded. The ties that reached tensile strength broke and lost the restraint of the core concrete column. The volume swelling of the concrete block was visible to the naked eye, mainly in the middle and upper parts. The crack width increased and the concrete surface blocks peeled off. The crack width decreased significantly when the crack propagation path passed through the tie region, indicating that the ties could effectively inhibit crack development. In addition, compared with UC, the integrity of CC was better after failure. The crack and the cross section of the concrete were approximately 45°.

**Figure 13:**
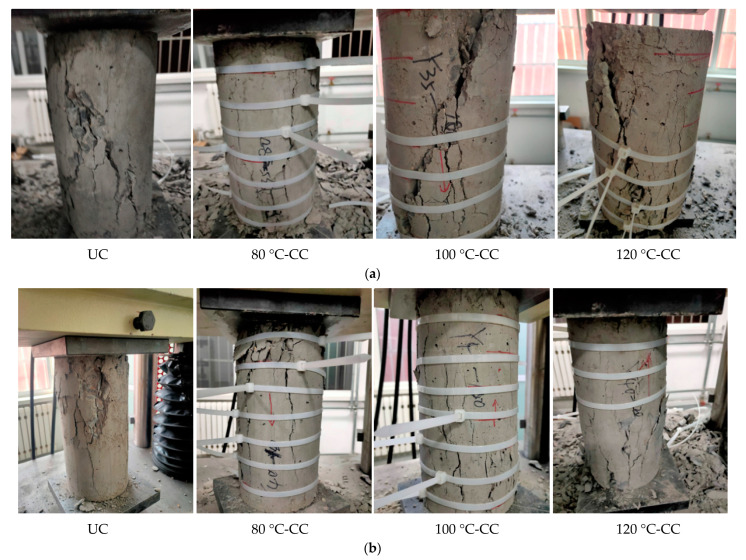

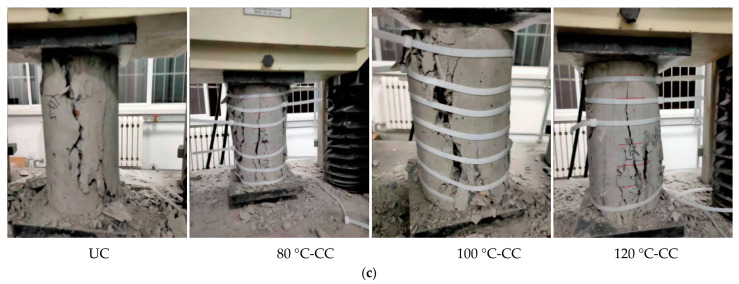
Damage to concrete: (**a**) C35 concrete, (**b**) C40 concrete, (**c**) C45 concrete.

During the test, the fracture of the ties occurred suddenly and rapidly. With an increase in the loading stress, the concrete column expanded, and the increase in the cross-sectional area led to an increase in the tensile stress. When the ties reached the maximum tensile strength, they lost their restraining ability and broke. The damage of concrete can be seen in Figure 13.

## 5. Numerical Results

**Figure 14:**
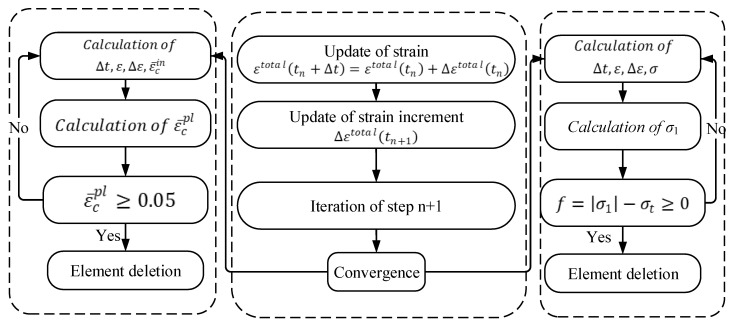
Flowchart of model yield failure.

Most of the research is limited to the constitutive or seismic research of confined concrete [26], and research on the damage evolution law of confined concrete is limited. Combined with the test results, in this section, a numerical simulation method was used to study the damage law of CC, providing a reference for the design and construction of preventive measures. As concrete columns in building structures mainly bear pressure, this section analyzes the compression damage law of concrete columns. Figure 14 is the flow chart of model yield failure.

### 5.1. Analysis of Damage Evolution Law of UC

**Figure 15:**
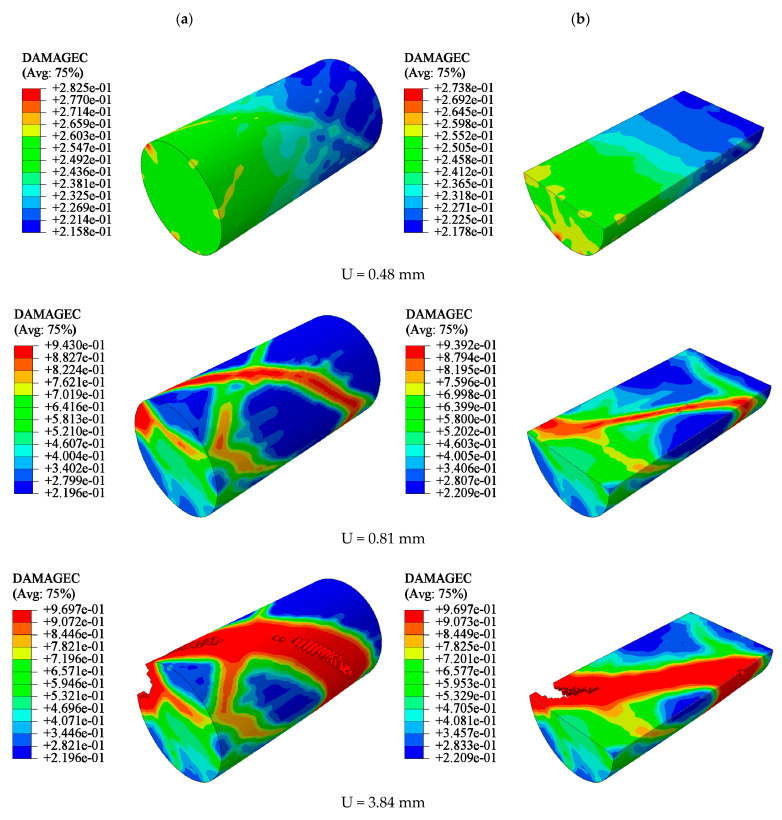

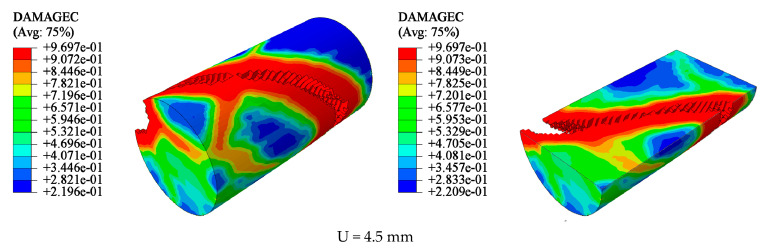
Damage process of unconfined CC (Y35): (**a**) uncut, (**b**) *X*-axis section.

A Y35 cylinder with a strength grade of C35 was selected as the study object. When the vertical displacement was U < 0.48 mm, the damage factor of the model was very small. The damage factor of each element of the column body exhibited a slight difference. In the range of U > 0.81 mm, with the increase in load, the damage factors of the column end and column body elements gradually increased. Thus, the cross-sectional area of the concrete increased. When the stress applied by the model was close to the peak stress, element damage appeared on the surface and inside the concrete model, first at the end of the column. Microcracks then appeared on the column. At a later stage of loading, the earlier microcracks gradually expanded and connected. Finally, the concrete model was sheared. The shear crack was approximately 45° to the concrete cross section. The shear cracks ran through the entire model (Figure 15).

The damage evolution law of the UC was at the early stage of loading; the damage factor of each part of the concrete did not differ significantly, and the stress-strain development was uniform. At the middle of loading, the damage factor of the concrete column body unit increased more than that of the other parts, and at this time, no damaged had appeared at the unit. At the later stage of loading, damage first appeared at the end of the column, and the column body exhibited “sporadic” damage. With an increase in load, sporadic damage gradually expanded and connected, and shear cracks formed. The cracks were wide and penetrated the column body at 45° to the column cross section. Cracks extended perpendicular to the principal stress and along the plane of the maximum shear stress, which was similar to the diagonal propagation of cracks in the structure.

### 5.2. Analysis of Damage Evolution Law of CC

**Figure 16:**
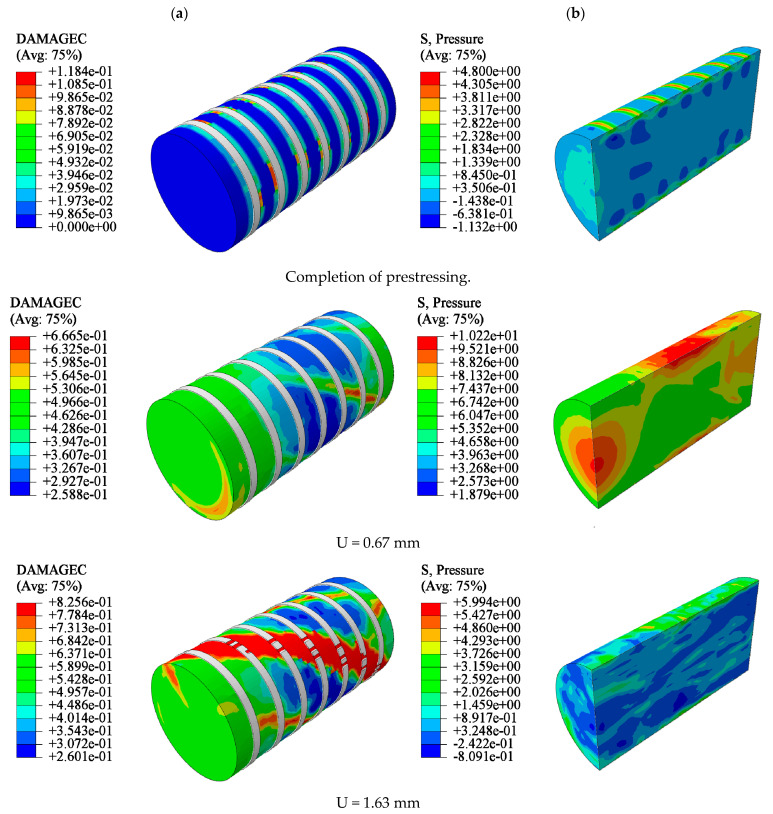

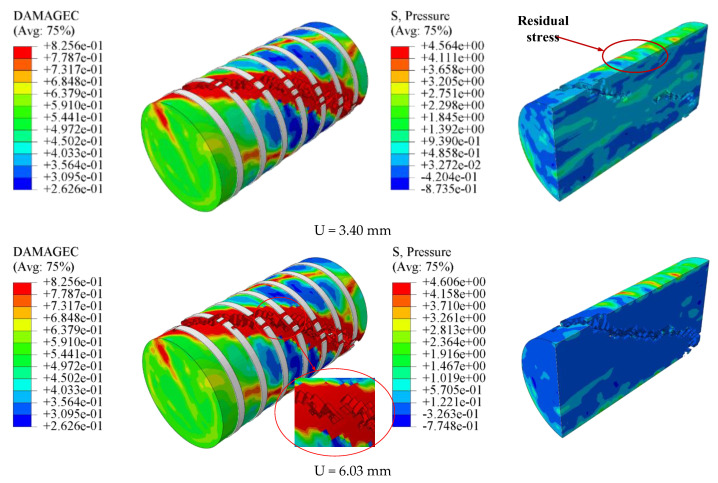
Damage process of confined CC (Y35-100): (**a**) uncut, (**b**) *X*-axis section.

Here, we take the Y35-100 specimen as an example. The CC was restrained by prestressing after its completion. When the loading displacement was small, the damage factors of the confined CCs were similar, and the lateral pressure slowly increased. An increase in the axial load of the confined CC resulted in an increase in the concrete volume. Owing to the interaction of forces, nylon ties bore tension from the concrete. When U = 1.63 mm, the tensile stress of the ties reached the tensile strength value and were then destroyed. However, there was residual restraint stress on the surface of the concrete restrained by ties. With an increase in displacement, the element reached the critical value of the damage and was removed. The cloud images show that the damage of the element located at the ties was lower, and that of the other parts of the model was greater. This is because the residual restraint stress on the surface of the model still restrained the crack propagation after the straps lost their restraint ability. Finally, shear failure occurred in the confined concrete. The shear crack was approximately 45° to the concrete cross section. Compared with the failure characteristics of UC, the failure development of CC was slow, its integrity was better, and the crack width was narrower (Figure 16).

The damage evolution law of CC was at the early stage of loading; the prestress provided by the ties effectively prevented the concrete unit damage factor from increasing, and the unit damage factor grew slowly. In the mid-loading period, the restraining ties reached the tensile strength, and lost their damage-restraining effect. Residual stresses existed on the concrete surface and inhibited crack expansion. At a later stage of loading, the column unit damage formed a shear crack. The crack was at an angle of 45° to the column section and penetrated the main body with a narrow crack width.

### 5.3. Comparison of Concrete Cylinder Test and Numerical Simulation Results

The reliability of the damage failure model was verified by the stress-strain curve and failure characteristics.

**Figure 17:**
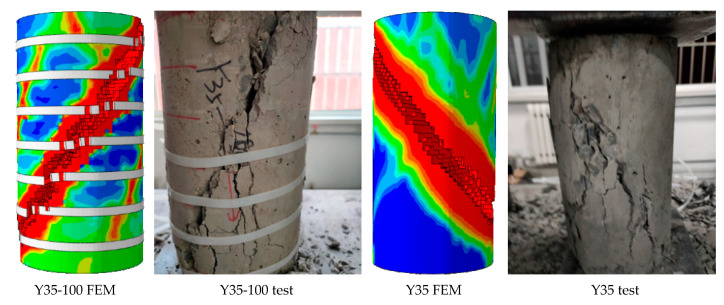
Validation of numerical and experimental results.

**Figure 18:**
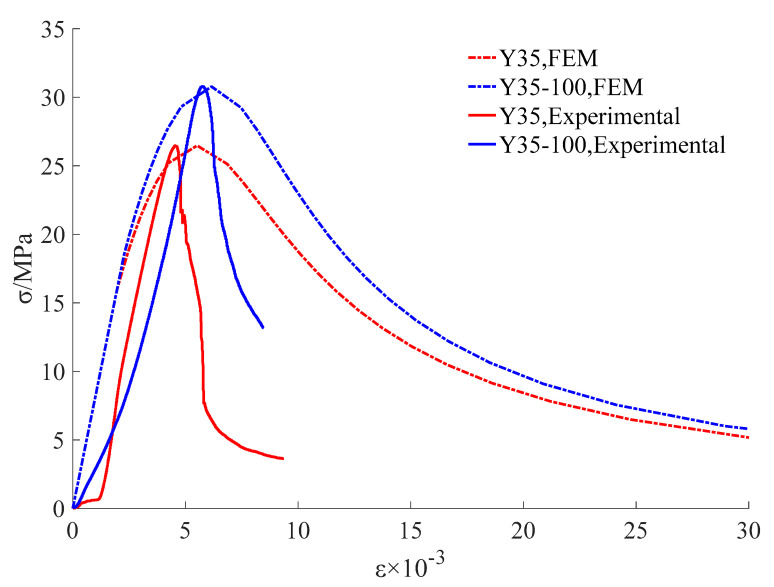
Stress-strain curve.

**Figure 19:**
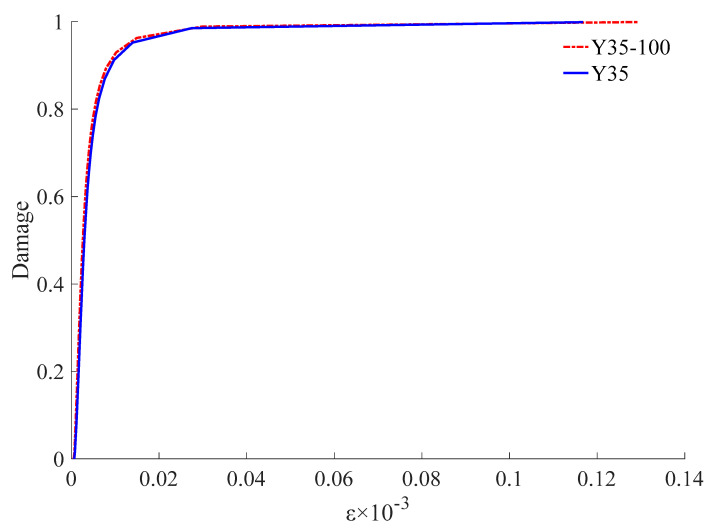
Damage-strain curve.

The numerical results were consistent with the test results (Figure 17, Figure 18 and Figure 19). The complete stress-strain curve of the uniaxial compression of concrete consisted of three stages: linear, hardening increasing, and softening decreasing. The slope of the linear rise section of concrete increased with an increase in concrete strength, and the slope and modulus of elasticity of the linear rise stage of CC were larger than those of UC. For ordinary concrete, the higher the strength, the steeper the softening section, the greater the brittleness, and the worse the ductility of the specimen. As shown in Figure 18, the softening and decreasing section of the stress-strain curve of Y35-100 was gentle and brittle compared to that of Y35. This shows that the prestressed nylon ties can improve the strength and ductility of the structure. In the linear rise section of the stress-strain curve, the stress intensity of CC was lower than that of UC under a given strain value. Thus, when the CC is confined to bear a vertical load in the early stage, the prestress can effectively resist an increase in the internal element stress of the material.

### 5.4. Damage Analysis of Nylon Ties

**Figure 20:**
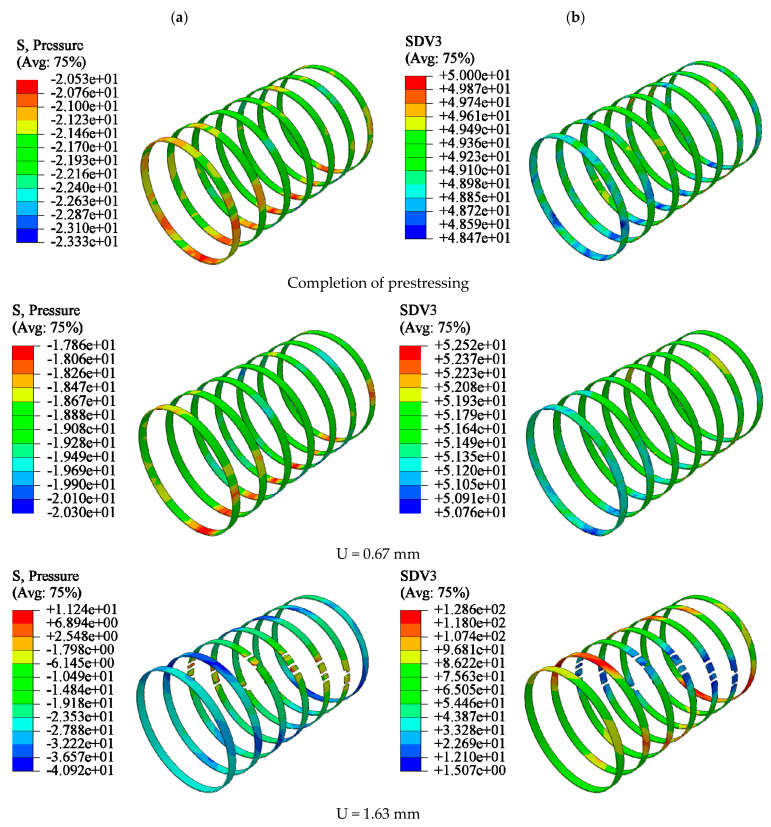

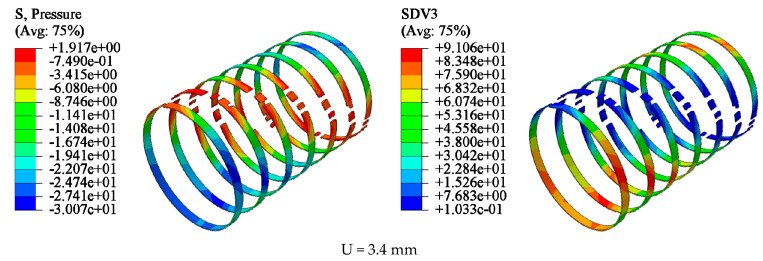
Fracture process of nylon ties: (**a**) lateral pressure (N), (**b**) maximum tensile stress (MPa).

To obtain the tensile stress distribution of the nylon ties, the calculation results of the maximum principal stress in each integral step state were assigned to the state variables in the VUMAT subroutine. They appear in the form of an SDV in the visualization module of ABAQUS software. SDV3 represents the maximum principal stress in each integral state (Figure 20).

**Figure 21:**
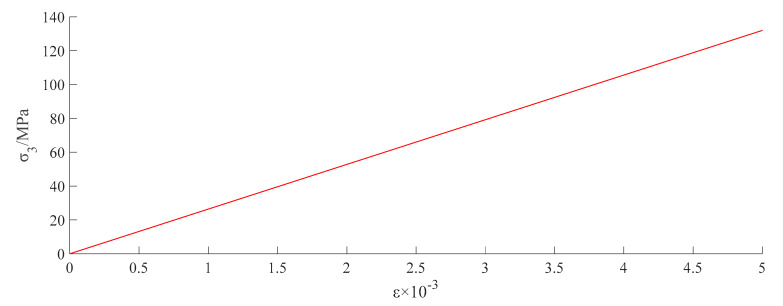
Tensile stress-strain curve of ties.

**Figure 22:**
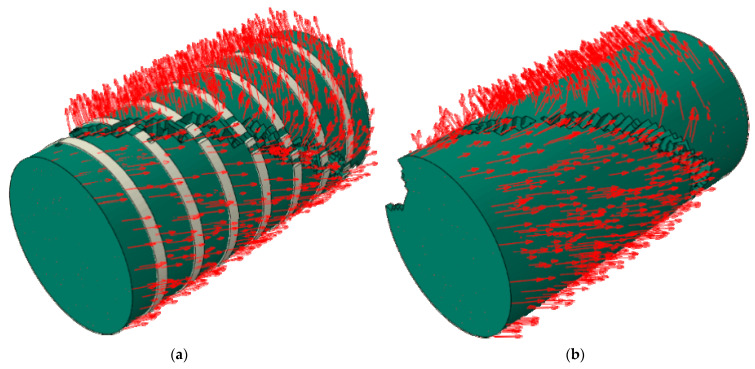
Displacement vector: (**a**) Y35-100, (**b**) Y35.

When the prestress was applied, the average lateral force and tensile stress of the ties were 20 N and 48.43 MPa, respectively. At this stage, the lateral restraint of the ties was caused by the cooling shrinkage of their own material. Before the vertical displacement of the concrete reached 1.63 mm, the lateral force on the ties increased slowly. The prestressing force exerted by the ties on the concrete restrained the expansion of concrete volume. When the cross-sectional area of the concrete increased to a certain extent, the prestressing force exerted by the ties on the concrete lost its effect. After this stage, the lateral force of the ties increased rapidly. When U = 1.63 mm, the ties in the middle of the CC first reached the tensile stress strength (127.64 MPa) and broke. The lateral force of the unbroken ties at the column end continued to increase. Under monotonic axial compression, bulging in the middle of the confined CC was more severe than that at both ends. The tensile stress of the tie in the middle was the largest and also the first to break (Figure 20). As can be seen from Figure 21, the tensile strain of the heated ties was proportional to the tensile stress, reflecting the hard and brittle characteristics. Figure 22 shows the simulated displacement vectors.

## 6. Conclusions

Based on the advantages of nylon, which include maintaining good strength after high-temperature cooling, this study explored the effect of prestressed nylon ties on the reinforcement of CCs. Combined with the test results, a numerical model was established to explore the damage evolution law of the CCs. The conclusions are as follows.

1. The tensile strength of the nylon ties decreased with increasing temperature, but the preload caused by the cold shrinkage of the ties increased. After high temperature cooling at 80 °C, 100 °C, and 120 °C, the tensile strengths of the tie were 138 MPa, 127.64 MPa, and 113.41 MPa, respectively, and the average pre-tightening forces generated by the ties were 27.82 kPa, 33.33 kPa, and 44.29 kPa, respectively.
2. The prestress produced by nylon ties with different heating temperatures owing to the decrease in temperature can enhance the bearing capacity, compressive strength, and initial elastic modulus of CCs; the higher the heating temperature, the higher the prestress and the more evident the restraint effect. Compared with the UC, the compressive strength of specimens with 80 °C, 100 °C, and 120 °C confinement schemes increased by 9.46%, 15.73%, and 20.06%, respectively.
3. The finite element software ABAQUS was used to establish the damage-failure model of the CC. The damage laws of confined CCs under uniaxial compression were analyzed from the aspects of maximum principal strain, equivalent plastic strain, and compression damage, which can effectively improve the ductility and elastic modulus of members. At the initial loading stage, the prestress applied by the ties effectively inhibited the volume expansion of the concrete, and the lateral pressure on the ties increased slowly. With the increase in load, the effect of the prestressed restraint decreased, and the growth rate of the lateral force of the tie belts accelerated. Combined with the results of the numerical simulation and test, the reliability of the damage model was verified, providing a theoretical reference for the study of the damage law of engineering reinforcements.

## Figures and Tables

**Table 1:** Grouping of specimens.

Concrete Strength	Number	Heating Temperature of Nylon Ties (Deviation: ±1 °C)	Binding Space of Nylon Ties	Specimen Size
C35	Y35-120	120 °C	40 mm	ϕ150 mm × 300 mm
	Y35-100	100 °C	40 mm	ϕ150 mm × 300 mm
	Y35-80	80 °C	40 mm	ϕ150 mm × 300 mm
	Y35	-	-	ϕ150 mm × 300 mm
C40	Y40-120	120 °C	40 mm	ϕ150 mm × 300 mm
	Y40-100	100 °C	40 mm	ϕ150 mm × 300 mm
	Y40-80	80 °C	40 mm	ϕ150 mm × 300 mm
	Y40	-	-	ϕ150 mm × 300 mm
C45	Y45-120	120 °C	40 mm	ϕ150 mm × 300 mm
	Y45-100	100 °C	40 mm	ϕ150 mm × 300 mm
	Y45-80	80 °C	40 mm	ϕ150 mm × 300 mm
	Y45	-	-	ϕ150 mm × 300 mm

**Table 2:** Performance of nylon ties.

Performance of Nylon Ties			
Physical properties	Density		1.14 kg/m^3^
	Glass transition temperature		55–58 °C
	Deformation rate	80 °C	0.10%
		100 °C	0.15%
		120 °C	0.19%
Mechanical properties	Tensile strength	80 °C	138 MPa
		100 °C	127.64 MPa
		120 °C	113.41 MPa
	Coefficient of linear expansion		1.14 × 10^−5^/°C
Heat resistance [16]	Melting point		252–265 °C
	Embrittlement temperature		−30 °C
	Continuous deformation resistance temperature		80–120 °C
	Specific heat capacity		1700 J/(kg·K)

**Table 3:** Prestress of radial winding (kPa).

Time	Heated Temperature of Nylon Ties		
	80 °C	100 °C	120 °C
1 min	26.07	37.27	42.59
2 min	35.43	48.62	57.34
4 min	44.37	52.14	66.52
8 min	44.41	52.19	66.68
16 min	44.41	52.19	66.68

**Table 4:** Prestress of circumferential winding (kPa).

Time	Heated Temperature of Nylon Ties		
	80 °C	100 °C	120 °C
1 min	7.89	11.76	14.06
2 min	9.43	12.94	16.12
4 min	11.10	14.31	17.76
8 min	11.24	14.46	17.89
16 min	11.24	14.46	17.89

## Data Availability

Not applicable.
